# Genetic boundaries delineate the potential human pathogen *Salmonella bongori* into discrete lineages: divergence and speciation

**DOI:** 10.1186/s12864-019-6259-z

**Published:** 2019-12-04

**Authors:** Xiaoyu Wang, Songling Zhu, Jian-Hua Zhao, Hong-Xia Bao, Huidi Liu, Tie-Min Ding, Gui-Rong Liu, Yong-Guo Li, Randal N. Johnston, Feng-Lin Cao, Le Tang, Shu-Lin Liu

**Affiliations:** 1 Systemomics Center, College of Pharmacy, and Genomics Research Center (State-Province Key Laboratories of Biomedicine-Pharmaceutics of China), Harbin Medical University, 157 Baojian Road, Harbin, 150081 China; 2 HMU-UCCSM Centre for Infection and Genomics, Harbin Medical University, Harbin, China; 3 Translational Medicine Research and Cooperation Center of Northern China, Heilongjiang Academy of Medical Sciences, Harbin, China; 4 Department of Medicine and Food Engineering, Harbin Labor Technician College, Harbin, China; 5 Department of Infectious Diseases, The First Affiliated Hospital, Harbin Medical University, Harbin, China; 6 Department of Biochemistry and Molecular Biology, University of Calgary, Calgary, Canada; 7 Department of Hematology, The First Affiliated Hospital, Harbin Medical University, Harbin, China; 8 Department of Ecosystems and Public Health, University of Calgary, Calgary, Canada; 9 Department of Microbiology, Immunology and Infectious Diseases, University of Calgary, Calgary, Canada

**Keywords:** *Salmonella*, Bacterial pathogens, Genomic divergence, Genetic boundary

## Abstract

**Background:**

*Salmonella bongori* infect mainly cold-blooded hosts, but infections by *S. bongori* in warm-blooded hosts have been reported. We hypothesized that *S. bongori* might have diverged into distinct phylogenetic lineages, with some being able to infect warm-blooded hosts.

**Results:**

To inspect the divergence status of *S. bongori*, we first completely sequenced the parakeet isolate RKS3044 and compared it with other sequenced *S. bongori* strains. We found that RKS3044 contained a novel T6SS encoded in a pathogenicity island-like structure, in addition to a T6SS encoded in SPI-22, which is common to all *S. bongori* strains so far reported. This novel T6SS resembled the SPI-19 T6SS of the warm-blooded host infecting *Salmonella* Subgroup I lineages. Genomic sequence comparisons revealed different genomic sequence amelioration events among the *S. bongori* strains, including a unique CTAG tetranucleotide degeneration pattern in RKS3044, suggesting non-overlapping gene pools between RKS3044 and other *S. bongori* lineages/strains leading to their independent accumulation of genomic variations. We further proved the existence of a clear-cut genetic boundary between RKS3044 and the other *S. bongori* lineages/strains analyzed in this study.

**Conclusions:**

The warm-blooded host-infecting *S. bongori* strain RKS3044 has diverged with distinct genomic features from other *S. bongori* strains, including a novel T6SS encoded in a previously not reported pathogenicity island-like structure and a unique genomic sequence degeneration pattern. These findings alert cautions about the emergence of new pathogens originating from non-pathogenic ancestors by acquiring specific pathogenic traits.

## Background

*Salmonella* bacteria are ubiquitous pathogens, with over 2600 serotypes documented to date [1]. Based on different levels of relatedness, the *Salmonella* bacteria are categorized into eight subgroups, e.g., Subgroups I, II, IIIa, IIIb, IV, V, VI and VII [2, 3]. Human salmonellosis is primarily caused by *Salmonella* Subgroup I lineages but may occasionally be elicited also by bacteria of the other subgroups, which usually infect cold-blooded hosts [4]. Bacteria of Subgroup V (also known as *Salmonella bongori*; see the dynamic changes of *Salmonella* taxonomy and nomenclature in previous publications [5, 6]) lineages are among the least likely human salmonellosis agents, partly due to their lack of *Salmonella* Pathogenicity Island 2, which is required for the bacteria to survive in phagocytes and invade the deep tissues of the host [7, 8]. However, *S. bongori* infections do occur in humans or other warm-blooded hosts [9–11], opening a question about whether certain *S. bongori* lineages with special genetic traits might have facilitated such infections. A first step toward answering this question is the revelation of the genetic differences among the *S. bongori* bacteria isolated from different host species. We postulate that certain genomic characteristics might be owned by some but not other phylogenetic lineages of *S. bongori*, and such lineage-specific characteristics may be identified through comparative genomic analyses. To start with, the core question here is whether *S. bongori* consist of genetically distinct lineages.

In a previous study, we found that the physical structure of bacterial genomes could remain highly conserved for hundreds of millions of years in evolution [12, 13], but in the meantime some subtle genomic features can unambiguously reflect the phylogenetic distinction between even very closely related bacteria [14, 15]. As such, genome structure analysis may provide objective and reliable parameters for differentiating bacteria based on their evolutionary relationships rather than according to any arbitrary standards. To prove this postulation, we recently profiled genomic characteristics among representative human-infecting *Salmonella* pathogens and demonstrated the existence of genetic boundaries that can be used to divide the *Salmonella* bacteria into clear-cut phylogenetic clusters [16–21].

The *Salmonella* bacteria were initially treated as individual species beginning from early 1880s primarily due to their distinct pathogenic features, such as *Salmonella typhimurium* causing gastroenteritis or *Salmonella typhi* causing typhoid fever in humans, and were differentiated by serotyping based on their different combinations of O and H antigens [22, 23]. As a result, the *Salmonella* species were represented each by an antigenic formula, such as *S. typhimurium* by 1,4, [5],12:i:1,2 or *S. typhi* by 9,12,[vi]:d:-. However, in the mid-1980s, all *Salmonella* species were re-classified into a single new species under the specific name *enterica* based on their close genetic relatedness. Consequently, the previous species became serovars of the only *Salmonella* species, *Salmonella enterica* [24]. Later, Subgroup V regained the scientific name of *Salmonella bongori* based on its greater genetic divergence from all other subgroups [25]. In this report, we use the pre-1980 *Salmonella* nomenclature for reasons as detailed in a previous publication [6].

Different from Subgroup I lineages that are mostly monophyletic serotypes, each having had a Latinized scientific name prior to the reclassification of all *Salmonella* species into the single species *enterica*, Subgroup V lineages all have the same scientific name *Salmonella bongori*. To date, more than 20 serotypes have been documented in *S. bongori*, but the precise phylogenetic relationships of the bacteria among the serotypes and the phyletic status within each of the serotypes remain largely unclear.

In this study, we completely sequenced and annotated the genome of a parakeet isolate of *S. bongori*, strain RKS3044, which is one of the *Salmonella* Reference Collection C strains kindly provided by Dr. Robert K. Selander (www.ucalgary.ca/~kesander), and compared it with other sequenced *S. bongori* strains that had different antigenic formulae or were isolated from cold-blooded hosts. Here we report our findings that the sequenced *S. bongori* strains have diverged into distinct lineages and, interestingly, strain RKS3044 had a novel cluster of T6SS-associated genes. Whereas we cannot conclude that the additional T6SS might be involved in the warm-blooded host invasion by RKS3044, our results show that *S. bongori* bacteria can be circumscribed into discrete phylogenetic clusters, each having a distinct set of genomic characteristics.

## Results

### Genomic comparisons of the completely sequenced *S. bongori* strains: RKS3044 representing a warm-blooded host pathogen

**Fig. 1:**
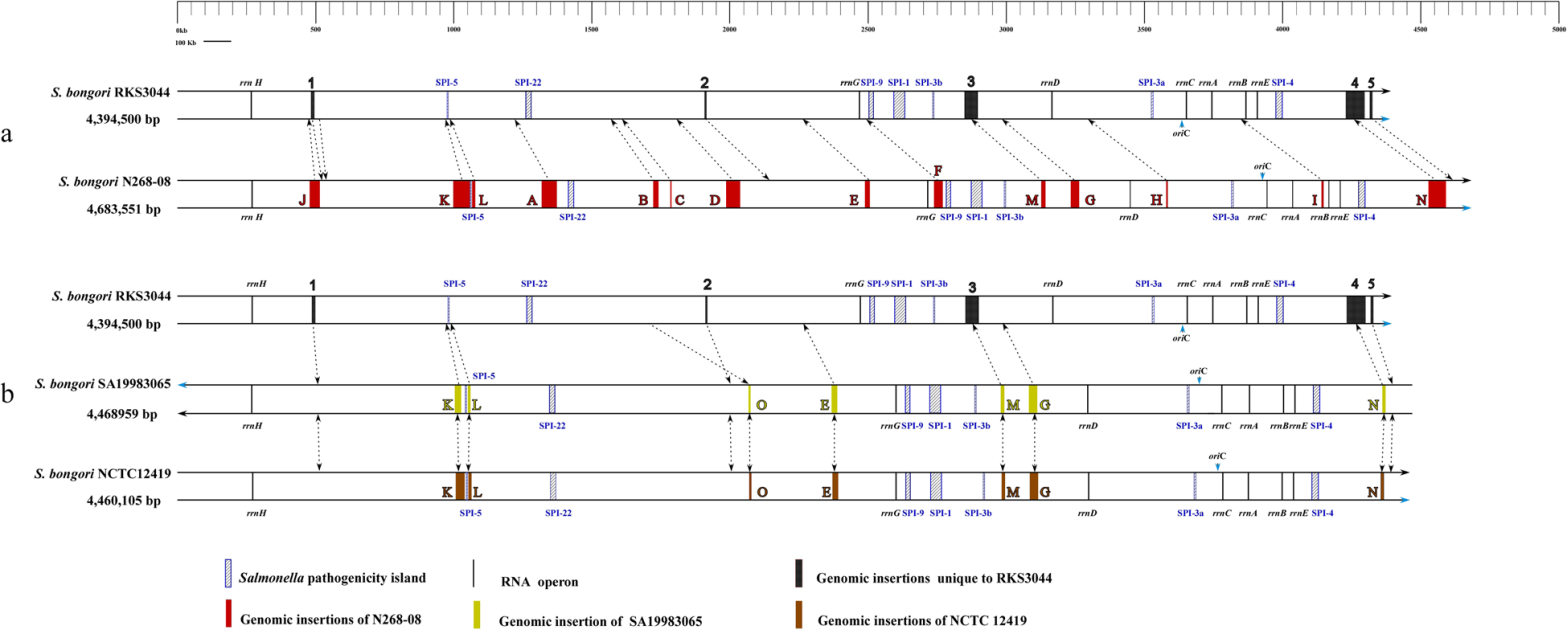
Genomic comparisons of RKS3044 with other completely sequenced *S. bongori* strains. **a**, comparison between RKS3044 and N268-08; **b**, comparisons of RKS3044 with SA19983065 and NCTC12419. The circular genomes are linearized here for the convenience of presentation, starting from *thrL*. Insertions with the size of 5 kb or up are shown here. Insertions unique to RKS3044 are indicated by numbers from 1 to 5. Insertions present in other *S. bongori* strains but absent in RKS3044 are indicated by capital letters from A to O. Dotted arrow show the homologous site of an insertion in other genomes that do not carry it. The information related to all genomic insertions shown in the figure can be found in Additional file 4: Table S2

For overall comparisons, we first completely sequenced *S. bongori* strain RKS3044. Its genome consists of a single chromosome of 4,394,500 bp (51.4% GC content), which has a perfectly balanced physical structure between *oriC* and *terC* like most other reported *Salmonella* genomes [13, 26, 27] (Additional file 1: Figure S1); detailed information on the genome is summarized in Table 1 and the distribution of genes into COGs functional categories is presented in Table 2. We compared RKS3044 with three previously sequenced *S. bongori* strains, including N268–08 [28], NCTC12419 [29] and SA19883065 (www.ucalgary.ca/~kesander), and draft genomic sequences of several other *S. bongori* strains (Additional file 3: Table S1). When the four complete genomes were aligned, we found remarkable differences among them, particularly in different sets of insertions such as *Salmonella* Pathogenicity Islands (SPIs), which make the genome sizes considerably different among them (Fig. 1 and Additional file 4: Table S2). Whereas strains NCTC12419 and SA19983065, both being 66:z41:-, are highly similar, RKS3044 (48:z41:-) and N268–08 (antigenic formula unknown) are very different from each other and both are different from the pair of NCTC12419/SA19983065 strains. Such fundamental genomic differences suggest phylogenetic divergence over long evolutionary times among these bacteria as discussed previously [17, 18, 20].

**Table 1:** General characteristics of the complete *S. bongori* RKS3044 genome

Attribute	Value	% of Total
Genome size (bp)	4,394,500	100% of total genome
DNA coding (bp)	3,859,991	87.84% of total genome
DNA G + C (bp)	2,258,648	51.40% of total genome
Total predicted genes	4121	87.84% of total genome
Protein coding genes	4017	97.48% of total genes
RNA genes	22	0.53% of total genes
Pseudo genes	77	1.87% of total genes
Genes in internal clusters	2847	69.09% of total genes
Genes with function prediction	3563	86.46% of total genes
Genes assigned to COGs	3066	74.40% of total genes
Genes with Pfam domains	2449	68.54% of total genes
Genes with signal peptides	378	9.17% of total genes
Genes with transmembrane helices	1023	24.82% of total genes
CRISPR repeats	2

**Table 2:** Categories of genes associated with 25 general COG functions

Code	Value	% of total ^a^	Description
J	174	0.57	Translation
A	1	0.03	RNA processing and modification
K	257	7.49	Transcription
L	132	3.85	Replication, recombination and repair
B	0	0.00	Chromatin structure and dynamics
D	31	0.90	Cell cycle control, mitosis and meiosis
Y	0	0.00	Nuclear structure
V	46	1.34	Defense mechanisms
T	152	4.43	Signal transduction mechanisms
M	215	6.27	Cell wall/membrane biogenesis
N	103	3.00	Cell motility
Z	0	0.00	Cytoskeleton
W	0	0.00	Extracellular structures
U	113	3.29	Intracellular trafficking and secretion
O	143	4.17	Posttranslational modification, protein turnover, chaperones
C	236	6.88	Energy production and conversion
G	324	9.45	Carbohydrate transport and metabolism
E	318	9.27	Amino acid transport and metabolism
F	81	2.36	Nucleotide transport and metabolism
H	144	4.20	Coenzyme transport and metabolism
I	82	2.39	Lipid transport and metabolism
P	182	5.31	Inorganic ion transport and metabolism
Q	47	1.37	Secondary metabolites biosynthesis, transport and catabolism
R	333	9.17	General function prediction only
S	316	9.12	Function unknown
–	1055	25.60	Not in COGs

^a^The total is based on the total number of protein coding genes in the annotated genome

### Phylogenetic relationships among the *S. bongori* bacteria

**Fig. 2:**
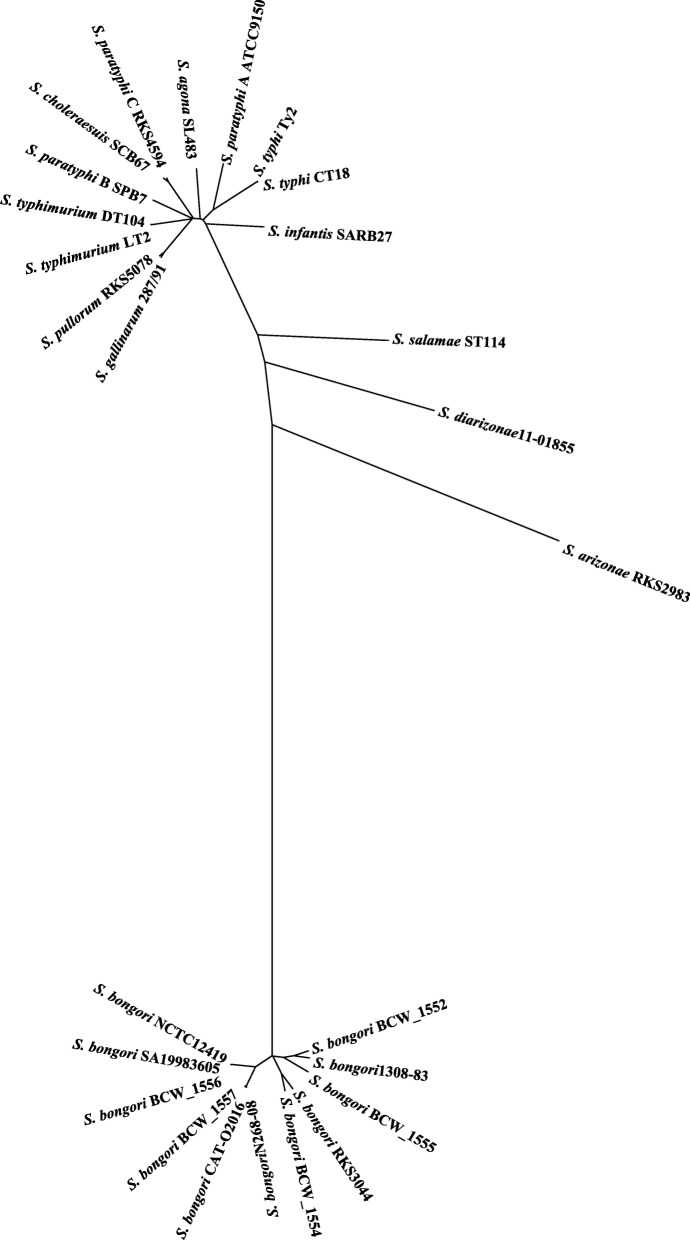
Phylogenetic tree of the *Salmonella* strains. The phylogenetic tree was constructed by genes common to the 26 *Salmonella* strains representing *Salmonella* subgroups I, II, IIIa, IIIb, and V

To estimate the population phylogenetic structure of *S. bongori*, we analyzed the sequenced *S. bongori* strains (see Additional file 3: Table S1) in comparison with representative strains of the other *Salmonella* subgroups (Additional file 5: Table S3). We concatenated 2804 genes common to the 26 analyzed strains and constructed a phylogenetic tree. We found that in general the *S. bongori* strains had similar genetic distances from one another as those seen among the Subgroup I strains (Fig. 2). For example, *S. bongori* strains RKS3044 and N268–08 had a genetic distance between them similar to that as between *S. typhi* and *S. typhimurium*, two serotypes that are currently categorized into the same taxonomic species but are in fact vastly different pathogens. As we have recently resolved *S. typhi* and *S. typhimurium* into different natural species based on the clear-cut genetic boundary (i.e., allelic distance) between them [6, 19], we wondered whether a genetic boundary may also have been formed between *S. bongori* strains RKS3044 and N268–08.

### Genetic boundaries delineating the *S. bongori* strains into clear-cut phylogenetic clusters

To evaluate the phylogenetic significance of the demonstrated genetic separation among the *S. bongori* strains, i.e., to determine whether the genetic divergence might be clear-cut (i.e., making the lineages clearly discrete) or continuous (i.e., showing a spectrum of gradual or continuous genetic differences without clear cut-offs among the “lineages”), we detected the percentages of the homologous genes that share identical nucleotide sequences (zero nucleotide degeneracy) between the bacteria (Additional file 2: Figure S2 and Additional file 6: Table S4). Based on the percentages, we categorized the *S. bongori* strains into three groups: high, from 85 to 100% (e.g., between N268–08 and BCW_1557 or CATO-2016, between NCTC12419 and SA19983605 or BCW_1556, etc.); medium, 20–35% (e.g., between RKS3044 and BCW_1554, between SA19983605 and CATO-2016, between 1308 and 83 and BCW_1552); low, lower than 20% and mostly around 10% (the majority of the analyzed *S. bongori* strains; Additional file 2: Figure S2). The “high” group resembles strains within *S. typhi*, which are highly cohesive as members of the same phylogenetic cluster [19]; the “medium” group resembles bacteria between *S. gallinarum* and *S. pullorum*, which have diverged into separate lineages for not long and hence remain highly related though with obvious genetic distinction between them [16]; the “low” group resembles bacteria between most *Salmonella* Subgroup I serotypes like *S. typhi* and *S. typhimurium*, which are circumscribed into discrete phylogenetic clusters of bacteria equivalent to natural species [6, 16, 19]. In this study, we did not find *S. bongori* strains that had the percentages between 35 and 85%, probably because the number of *S. bongori* strains analyzed here was not large enough to cover sufficient ranges of the genetic divergence, although our previous work has demonstrated that the “intermediate percentages”, such as those around 40% between *S. gallinarum* and *S. pullorum* [16], is indicative of phylogenetic divergence of the compared bacteria into distinct species.

### Genes involved in pathogenesis and virulence: different levels of evolutionary conservation between *S. bongori* and other *Salmonella* subgroups

**Fig. 3:**
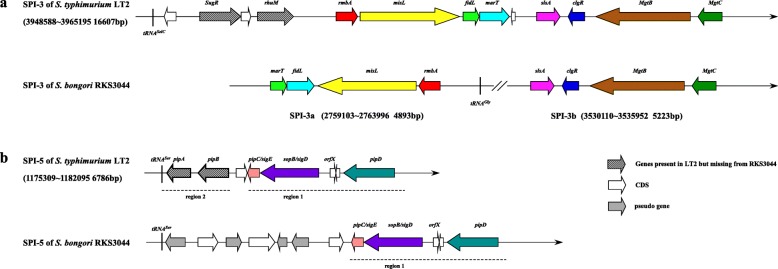
Different structures of SPI-3 and SPI-5 between *S. bongori* RKS3044 and *S. typhimurium* LT2. **a**, comparison of SPI-3 between *S. typhimurium* LT2 and *S. bongori* RKS3044; **b**, comparison of SPI-5 between *S. typhimurium* LT2 and S. bongori RKS3044. Homogenous genes are drawn in the same color. Note that genes missing from RKS3044 SPIs relative to LT2 are in gray grid

RKS3044 possesses all SPIs previously reported in *S. bongori*, including SPIs-1, 3, 4, 5, 9 and 22, which have various levels of structural or sequence similarities as exemplified by SPIs-3 and 5 between *S. bongori* and *S. typhimurium* LT2 (Fig. 3). By search against VFDB (Virulence Factors Pathogenic Bacteria, www.ncbi.nlm.nih.gov), we identified most of the previously reported *S. bongori* virulence genes in RKS3044 strain, except some of the genes encoding T3SS effector proteins (Additional file 7: Table S5). In addition to the known SPIs, we identified a novel genomic island in RKS3044, which we temporarily designate as SPI-RKS3044 (Insertion 3 in Fig. 1). This 48 kb SPI was RKS3044-specific, flanked at the upstream end by a tRNA^Phe^ gene and having fluctuated GC contents, an indication of mosaic DNA segments of different evolutionary origins.

### A new type VI secretion system in SPI-RKS3044

**Fig. 4:**
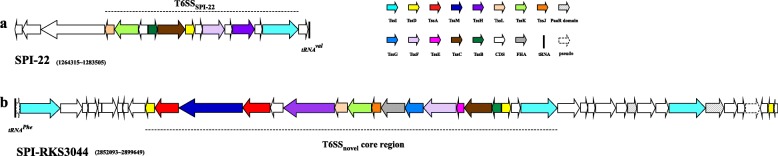
Gene organization of T6SS gene clusters in *S. bongori* RKS3044. **a**, SPI-22; **b**, SPI-RKS3044. Homologous genes are in the same color

Within SPI-RKS3044, we annotated a new Type VI secretion system (T6SS) at genomic location spanning nucleotides 2,861,308~2,886,179. Like other *S. bongori* strains, RKS3044 also has a T6SS in SPI-22. Therefore RKS3044 carries two T6SSs (Additional file 8: Table S6), but the two T6SS clusters have very different structures (Fig. 4), suggesting distinct evolutionary histories and different roles in bacterial pathogenesis between the two T6SSs. The T6SS in SPI-22 (Fig. 4a) has homologs encoding an Hcp-like protein (N643_RS05930) and a VgrG protein (N643_RS05960), which are both required for T6SS apparatus functionality; cytoplasmic proteins VipA (N643_RS05920) and VipB (N643_RS05925), which interact directly and are required for intracellular growth in macrophages; and other essential components such as DotU (N643_RS05890), ImpA (N643_RS05895), gp25-like protein (N643_RS05935) and VasA (N643_RS05940). However, in the SPI-22 T6SS we did not find homologs encoding COG0542 (ClpV), COG3521 (SciN) or COG3523 (IcmF) components that were present in previously reported SPI-22 T6SSs [29], which are usually required for the T6SS functionality. The novel T6SS in the 48 kb SPI-RKS3044 (designated T6SS_novel_) carries many more genes than the SPI-22 T6SS, covering nucleotides 2,852,331–2,899,649 (Fig. 4b), with structural similarity to the T6SS in SPI-19 (T6SS_SPI-19_) of *Salmonella* Subgroup I serotypes, such as *S. dublin, S. weltevreden, S. agona* and *S. gallinarum* [30].

### Phylogenetic analyses of T6SS gene clusters

**Fig. 5:**
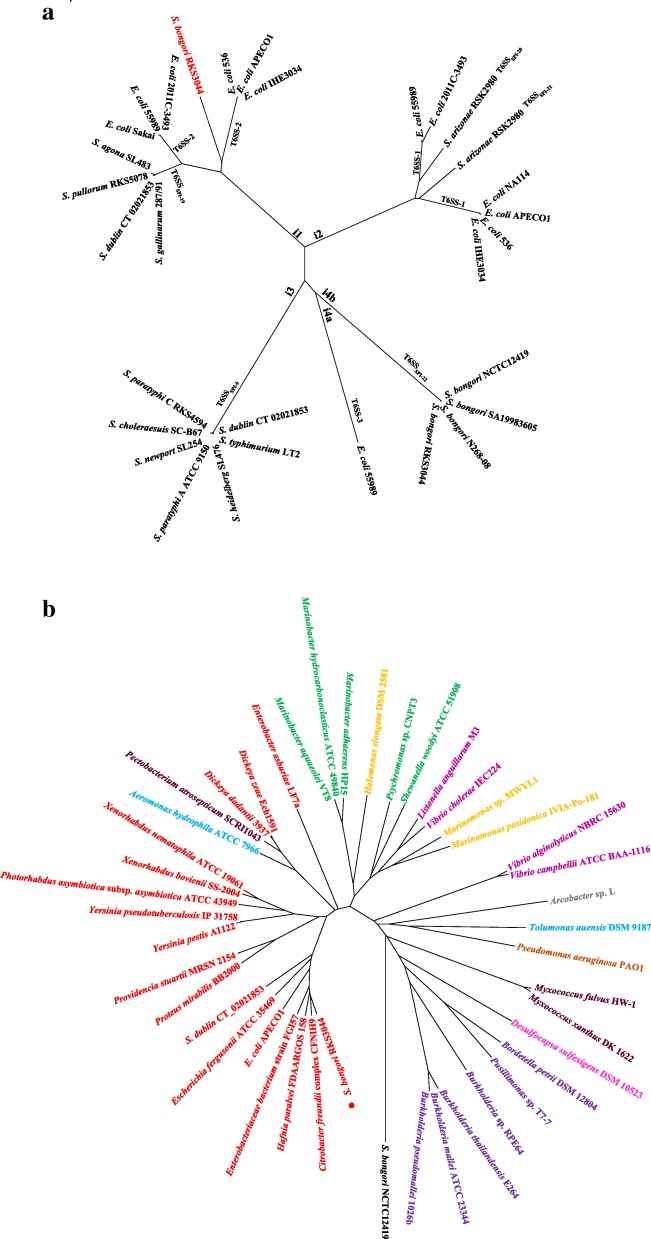
Evolutionary analysis of *S. bongori* RKS3044 T6SS_novel_. **a** comparison between T6SS_novel_ and T6SSs in selected *Salmonella* and *E. coli* lineages; the neighbor-joining tree was constructed from concatenated TssB and TssC protein sequences. **b** comparison between T6SS_novel_ and Type i1 T6SSs in selected Proteobacteria strains; the neighbour-joining tree was calculated from concatenated protein sequences of TssB, C, E, F, G, H, J, K and M, with the *S. bongori* NCTC12419 T6SS_SPI-22_ as outgroup

To assess the evolutionary relationships of the T6SSs in RKS3044 with those in closely related bacteria, we constructed a phylogenetic tree on concatenated TssB and TssC protein sequences of T6SS_novel_ and T6SS_SPI-22_ of RKS3044 and those from selected *E. coli* and *Salmonella* lineages. Previously, the *E. coli* T6SS gene clusters had been categorized into three distinct phylogenetic groups based on different levels of structural and sequence similarities: T6SS-1, T6SS-2 and T6SS-3, corresponding to Type i2, i1 and i4b, respectively [31–33]. On the phylogenetic tree, the *E. coli*-associated T6SSs and *Salmonella*-associated SPI-T6SSs were mixed together among five main branches, with the RKS3044 T6SS_novel_ appearing on the type i1 branch (Fig. 5a). This finding proves the horizontal acquisition nature of T6SSs. When we focused the phylogenetic analysis on T6SS_novel_ and other type i1 T6SSs in bacteria of 30 Proteobacteria, using the concatenated protein sequences of TssB, C, E, F, G, H, J, K and M, we found that RKS3044 T6SS_novel_ was clustered with T6SSs of other *Enterobacteriaceae* bacteria and was, interestingly, more closely related to that of *Citrobacter freundii* than to the *E. coli* and *Salmonella*- associated T6SSs (Fig. 5b and Additional file 9: Table S7).

### Genomic divergence by DNA sequence amelioration during evolution for adaptation as probed by unique CTAG degeneration patterns

RKS3044 and the other *S. bongori* strains form distinct phylogenetic lineages. We thus anticipated independent accumulation of genomic variations in these compared bacteria, including not only distinct sets of genomic insertions such as SPI-RKS3044 or T6SS_novel_ encoded within it, but also independent nucleotide substitutions such as the degeneration of some highly conserved short nucleotide sequences [17, 18, 20]. Our hypothesis was that, following the acquisition of certain peculiar traits such as the ability to expand host range, genomic DNA sequence amelioration may ensue to become better adapted to the new selection pressures, as seen in the divergence between the closely related *Salmonella* lineages *S. paratyphi* C and *S. choleraesuis* [34]. To robe such hypothesized genomic sequence amelioration events, we profiled the tetranucleotide sequence CTAG in the completely sequenced *S. bongori* strains and conducted systematic comparisons among the bacteria, focusing on its genomic location and degeneration patterns. We found that the CTAG sequence had different genomic distributions among the *S. bongori* lineages and the divergence patterns were consistent with the phylogenetic clustering of the bacteria, suggesting that the lineages have diverged into different phylogenetic and ecological positions for long evolutionary times, so they do not have much chances to freely exchange their genetic materials (Additional file 10: Table S8).

## Discussion

Among all documented bacteria to date, pathogenic species are a tiny portion but some of them are deadly, such as the typhoid agent *S. typhi*. Although genetic events involved in transforming a benign bacterial ancestor to a pathogen have been richly documented, a general picture about the origin of pathogenic bacterial species is lacking. Based on our previous findings that the ubiquitous *Salmonella* lineages, which are descendants of a common ancestor giving rise to *Escherichia* and *Salmonella* about 120–160 million years ago [35–37], all have their own unique sets of laterally acquired genes and genomic sequence amelioration patterns [12, 17, 38–40], we proposed the Adopt-Adapt model of bacterial speciation, with the “adopted” lateral genes diverting the direction of evolution and the ensuing genomic sequence amelioration for “adaptation” to accept the adopted genes and become increasingly fit to the new niche, e.g., a new host or environment [6, 26, 41]. The newly speciated bacterial lineage would continue accumulating further genomic variations independently and eventually become established in a separate gene pool isolated by a genetic boundary from others [16]. To prove whether the warm-blooded host isolate *S. bongori* RKS3044 might represent a novel natural species, we compared it with previously sequenced *S. bongori* strains to identify the hypothesized unique set of laterally adopted genes and adaptive sequence amelioration events in RKS3044 and to determine whether genetic boundaries might have been formed between RKS3044 and other *S. bongori* strains.

*S. bongori* bacteria have diverged from the other *Salmonella* lineages for ca. 40–63 million years [42] and they still form a tight phylogenetic cluster with obvious genetic distances from all other *Salmonella* subgroups [13, 25, 29]. As the extant *Salmonella* representative that first diverged from the common ancestor with *E. coli*, *S. bongori* may provide information about the evolutionary processes from a benign bacterial ancestor to the diverse *Salmonella* pathogens. The emergence of warm-blooded host infecting agent from the well-established cold-blooded host pathogen alerts the potential of *S. bongori* to become a common human pathogen.

We focused on comparisons between *S. bongori* RKS3044 and other sequenced *S. bongori* strains to reveal their possible genomic differences. We found that RKS3044 shared most of the virulence genes with the previously sequenced *S. bongori* strains and also lacked SPI-2, but on the other hand contained a novel T6SS (T6SS_novel_) encoded in a new SPI (SPI- RKS3044) identified in this study. As T6SS_novel_ shares significant structural and sequence similarity with SPI-19 T6SS, which is required for survival the macrophages and for efficient colonization into deep tissues of warm-blooded hosts [43–45], we postulated its evolutionary roles to divert a lineage of *S. bongori* toward infecting warm-blooded hosts. If so, according to the hypothesized Adopt-Adapt model of bacterial speciation, genomic analysis should reveal a unique nucleotide sequence amelioration pattern [17, 18, 20], distinct from those of all previously reported *S. bongori* strains. When we profiled the tetranucleotide sequence CTAG in the genome of RKS3044 and conducted comparisons with the other sequenced *S. bongori* strains, we found that the patterns were unique for RKS3044 and strain N268–08; strains NCTC12419 and SA19983065 shared another special pattern (Additional file 10: Table S8). As NCTC12419 and SA19983065 belong to the same serotype (66:z41:-) that is different from the antigenic formula of RKS3044 (48:z41:-), it seemed that the serotype situation in *S. bongori* might be similar to that of *Salmonella* Subgroup I lineages, in which most serotypes are monophyletic and the monophyletic serotypes correspond to natural species [6]. To prove this, we needed to resolve genetic boundaries among them and eventually found that the *S. bongori* serotypes were indeed separated into discrete phylogenetic clusters by large allelic distances similar to those of *Salmonella* Subgroup I lineages (Additional file 6: Table S4 and Additional file 1: Figure S1). These findings are consistent with our previous reports that bacteria of a monophyletic *Salmonella* lineage, such as *S. typhimurium, S. typhi, S. gallinarum* or *S. pullorum,* collected from a wide range of geographical, temporal or spatial spans, have a common genome structure or share the same gene pool as reflected by the genetic boundary that circumscribe them together and separate the monophyletic *Salmonella* lineages into discrete phylogenetic clusters [17, 18, 20, 46].

The concept of natural species for bacteria is based on the notion that bacteria of a monophyletic *Salmonella* lineage collected from a wide range of geographical, temporal or spatial spans have high percentages of homologous genes with zero sequence degeneracy, reflecting dynamic processes of clonal expansion to eliminate less fit subpopulations. On the other hand, bacteria of different *Salmonella* lineages, no matter how closely related they might be from one another, such as between *S. gallinarum* or *S. pullorum*, have low percentages of homologous genes with zero sequence degeneracy, since they as distinct lineages have diverged for a long evolutionary time and in the process accumulated mutations independently. Notably, there are usually broad windows between the “high” and “low” percentages (higher than 70% vs lower than 20%, without intermediates between 70 and 20%, with rare exceptions) [17, 18, 20, 46], which would make the allelic distance an applicable parameter to define and delineate bacteria into natural species. The method we used can effectively profile the nucleotide sequence information and the results can be evaluated by investigators of different laboratories. The low percentages of homologous genes with zero sequence degeneracy between different *Salmonella* lineages can be interpreted to be the consequences of independent accumulation of nucleotide variations by the bacteria over a long period.

To summarize, our results in this research show that *S. bongori* consist of discrete phylogenetic groups corresponding to the individual serotypes, which are equivalent to natural species. Whereas all sequenced *S. bongori* strains have a T6SS in SPI-22, strain RKS3044 has an additional T6SS in SPI- RKS3044. As the SPI-22 T6SS in RKS3044 is defective but the SPI-RKS3044 T6SS resembles the functional SPI-19 T6SS of the warm-blooded host infecting *Salmonella* Subgroup I lineages, it is possible that acquisition of the SPI-19-like T6SS might have facilitated the evolution of a branch of *S. bongori* represented by RKS3044 toward infecting warm-blooded hosts. A unique genomic sequence amelioration profile as reflected by the distinct CTAG tetranucleotide degeneration pattern from even very closely related *S. bongori* lineages suggests independent accumulation of genomic variations and the detection of a large allelic distance between RKS3044 and other *S. bongori* lineages/strains supported this postulation. Our study alerts cautions about the emergence of new pathogens originating from non-pathogenic ancestors, so preventive strategies are necessary to minimize such risks.

## Conclusions

The warm-blooded host-infecting *S. bongori* strain RKS3044 has distinct genomic features from other *S. bongori* strains, including a novel T6SS encoded in a previously not reported pathogenicity island-like structure and a unique genomic sequence degeneration pattern. These findings provide new support to the model of bacterial speciation to become pathogens.

## Methods

### Bacterial strains, growth conditions and DNA isolation

All bacterial strains used in this study were obtained from *Salmonella* Genetic Stock Center (SGSC; www.ucalgary.ca/~kesander) and cultured at 37 °C in LB broth or on LB agar plates. DNA was isolated from the bacterial cells by the CTAB (Cetyltrimethyl ammonium bromide) bacterial genomic DNA isolation method, purified and eluted by the QIAGEN DNA purified Kit (Qiagen Germany).

### Genome sequencing, assembly and annotation

The genome of *S. bongori* RKS3044 was sequenced by two sequencing platforms (SOLiD 3.0 and Illumina HiSeq 2000) according to the manual for the instrument. This work was initiated with SoLid time and we then added the HiSeq data to finish the sequencing project. Sequence data from the two methods were assembled by the velvet v1.2.09 software. We used the PFGE techniques to create a structural framework to align the contigs of SoLid and HiSeq short reads. The assembled scaffolds contained several gaps, which were closed by PCR amplification and ABI3730 sequencing. Eventually, we verified the correctness of the finished genome by PFGE based on cleavage data of XbaI, AvrII and SpeI. Genes were annotated using NCBI Prokaryotic Genome Automatic Annotation Pipeline (PGAAP) [47]. Additional analysis of gene prediction and annotation was supplemented using the IMG platform [48]. The graphical circular map of the *S. bongori* RKS3044 genome was generated with the CGviewer software.

### Comparative genome analysis

We concatenated the core genes common to the bacterial strains analyzed in this study and conducted comparisons using the Basic Local Alignment Search Tool (BLAST) with the parameters set at > 70% DNA identity and > 70% gene length to categorize genes into common genes. Individual orthologous sequences were aligned by the MAFFT program [49]. The phylogenetic trees of the genomes and T6SS loci were structured using the Neighbor-Joining method [50] in MEGA6 [51] by 1000 bootstrap replicates. The evolutionary distances were computed using the p-distance method [52]. To identify the virulence factor genes in RKS3044, we performed a BLAST search of whole RKS3044 ORFs against the virulence factor protein sequences of core dataset listed in VFDB [53] with an e-value of 1e-5. Genes encoding the T6SS were identified using the SecReT6 database [54].

For comparative analysis, genome sequences of representative *Salmonella* and *E. coli* strains were downloaded from the NCBI website (Additional file 5: Table S3).

## Supplementary information

**Additional file 1: Figure S1.** Graphical map of the *S. bongori* RKS3044 genome. From the outside to the center: genes on forward strand (color by COG categories), genes on reverse strand (color by COG categories), GC content, and GC skew. The map was generated with the CGviewer software.

**Additional file 2: Figure S2.** Genomic comparison among the *Salmonella bongori s*trains. Sequences common to eleven strains were concatenated and pair-wise aligned for the number of genes that have 100% sequence identity.

**Additional file 3: Table S1.** Sequenced *S. bongori* strains used for genomic comparisons in this study.

**Additional file 4: Table S2.** Profiles of genomic insertions in the completely sequenced *S. bongori* strains.

**Additional file 5: Table S3.** *Salmonella* and *E. coli* strains included in the comparative analysis.

**Additional file 6: TableS4.** Percentages of homologous genes that share identical nucleotide sequences between pairs of the bacteria compared.

**Additional file 7: Table S5.** Comparison of virulence gene profiles between *S. bongori* strains RKS3044 and NCTC12419.

**Additional file 8: Table S6.** Annotation of *S. bongori* RKS3044 SPI-22 and SPI-RKS3044 genes.

**Additional file 9: Table S7.** Bacterial strains used for comparative analysis of T6SS gene clusters.

**Additional file 10: Table S8.** CTAG profiles in completely sequenced *S. bongori* lineages/strains.

## Data Availability

The datasets generated and/or analysed during the current study are available in the NCBI website repository, (www.ncbi.nlm.nih.gov/ncbisearch/), with the accession number: *S. bongori* NCTC12419 (NC_015761), *S. bongori* N268–08 (NC_021870), *S. bongori* RKS3044 (NZ_CP006692), *S. bongori* SA19983605 (NZ_CP022120), *S. bongori* 1308–83 (NZ_MXLD01000001), *S. bongori* BCW_1557 (NZ_MXOC01000001), *S. bongori* BCW_1556 (NZ_MXOD01000001), *S. bongori* BCW_1555 (NZ_MXOE01000001), *S. bongori* BCW_1554 (NZ_MXOF01000001), *S. bongori* BCW_1552 (NZ_MXOH01000001), *S. bongori* CATO-2016 (NZ_NAPQ01000001), *S. typhi* Ty2 (NC_004631), *S. typhi* CT18 (NC_003198), *S. typhimurium* LT2 (NC_003197), *S. typhimurium* DT104 (NC_022569), *S. pullorum* RKS5078 (NC_016831), *S. gallinarum* 287/91 (NC_011274), *S. parayphi* A ATCC 9150 (NC_006511), *S. parayphi* B SPB7 (NC_010102), *S. parayphi* C RKS4594 (NC_012125), *S. choleraesuis* SC-B67 (NC_006905), *S. infantis* SARB27(CM001274), *S. agona* L483 (NC_011149), *S. arizonae* 62:z36:- RSK2983 (NZ_CP006693), *S. diarizonae* 11–01855 (NZ_CP011288), *S. salamae* 57:z29:z42 St114 (NZ_CP022467), *S. arizonae* 62:z4,z23:- RSK2980 (NC_010067), *S. heidelberg* SL476 (NC_011083), *S. dublin* CT_02021853 (NC_011205), *E. coli* O157:H7 Sakai (NC_002695), *E. coli* O104:H4 2011C-3493 (NC_018658), *E. coli* 55989 (NC_011748), *E. coli* 536 (NC_008253), *E. coli* IHE3034 (NC_017628), *E. coli* NA114 (NC_017644). All data generated or analysed during this study are included in this published article and its supplementary information files.
