# Supplementary figures and images for: Genetic boundaries delineate the potential human pathogen Salmonella bongori into discrete lineages: divergence and speciation
Source: BMC Genomics. 2019 Dec 4;20:930. doi: 10.1186/s12864-019-6259-z (PMC6894293; doi:10.1186/s12864-019-6259-z)

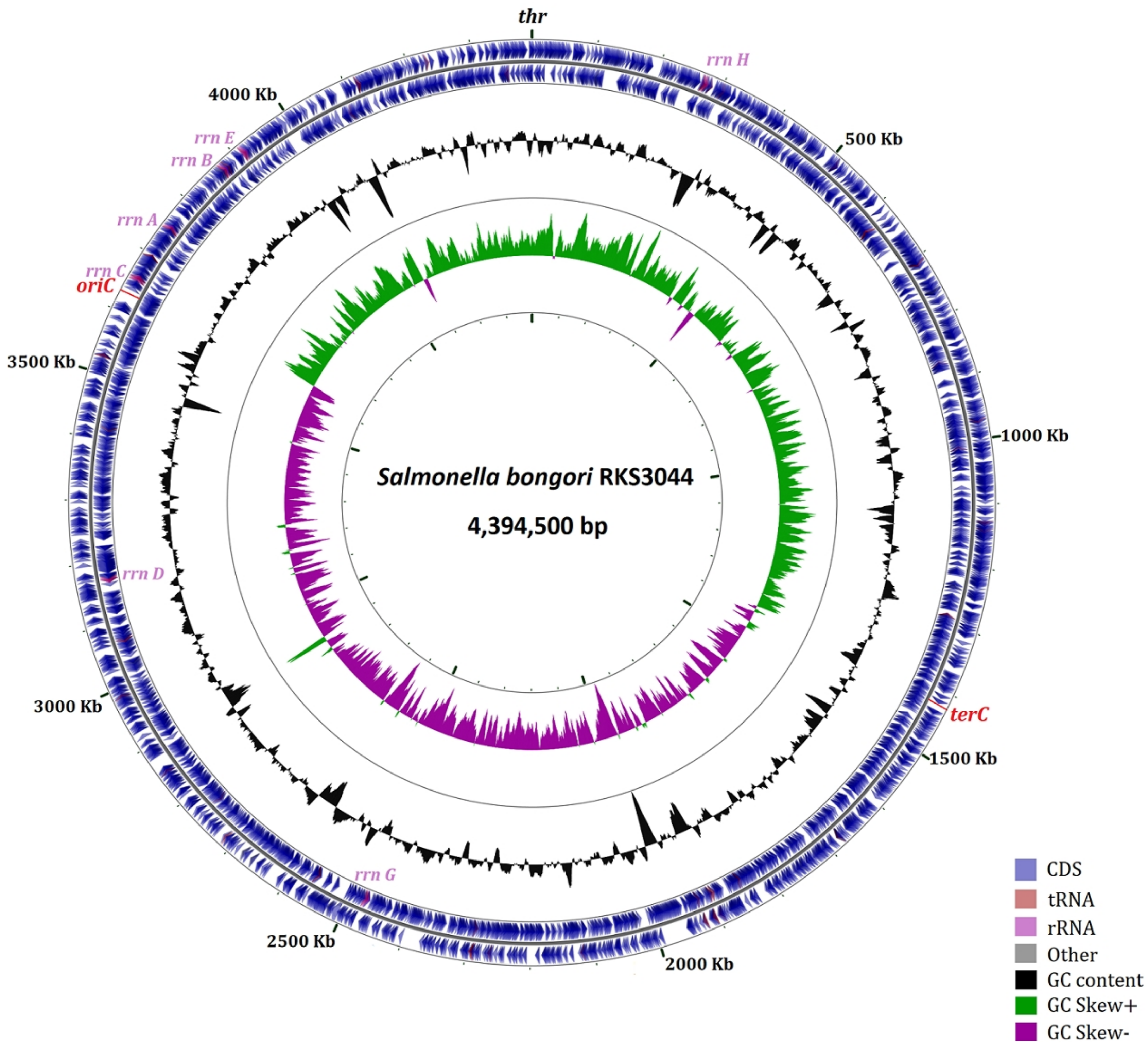

Supplement: Supplementary file 1 — Additional file 1: Figure S1. Graphical map of the S. bongori RKS3044 genome. From the outside to the center: genes on forward strand (color by COG categories), genes on reverse strand (color by COG categories), GC content, and GC skew. The map was generated with the CGviewer software. [file 12864_2019_6259_MOESM1_ESM.pdf]
