# Supplementary material for: Genetic boundaries delineate the potential human pathogen Salmonella bongori into discrete lineages: divergence and speciation
Source: BMC Genomics. 2019 Dec 4;20:930. doi: 10.1186/s12864-019-6259-z (PMC6894293; doi:10.1186/s12864-019-6259-z)
Supplement: Supplementary file 2 — Additional file 2: Figure S2. Genomic comparison among the Salmonella bongori strains. Sequences common to eleven strains were concatenated and pair-wise aligned for the number of genes that have 100% sequence identity. [file 12864_2019_6259_MOESM2_ESM.pdf]

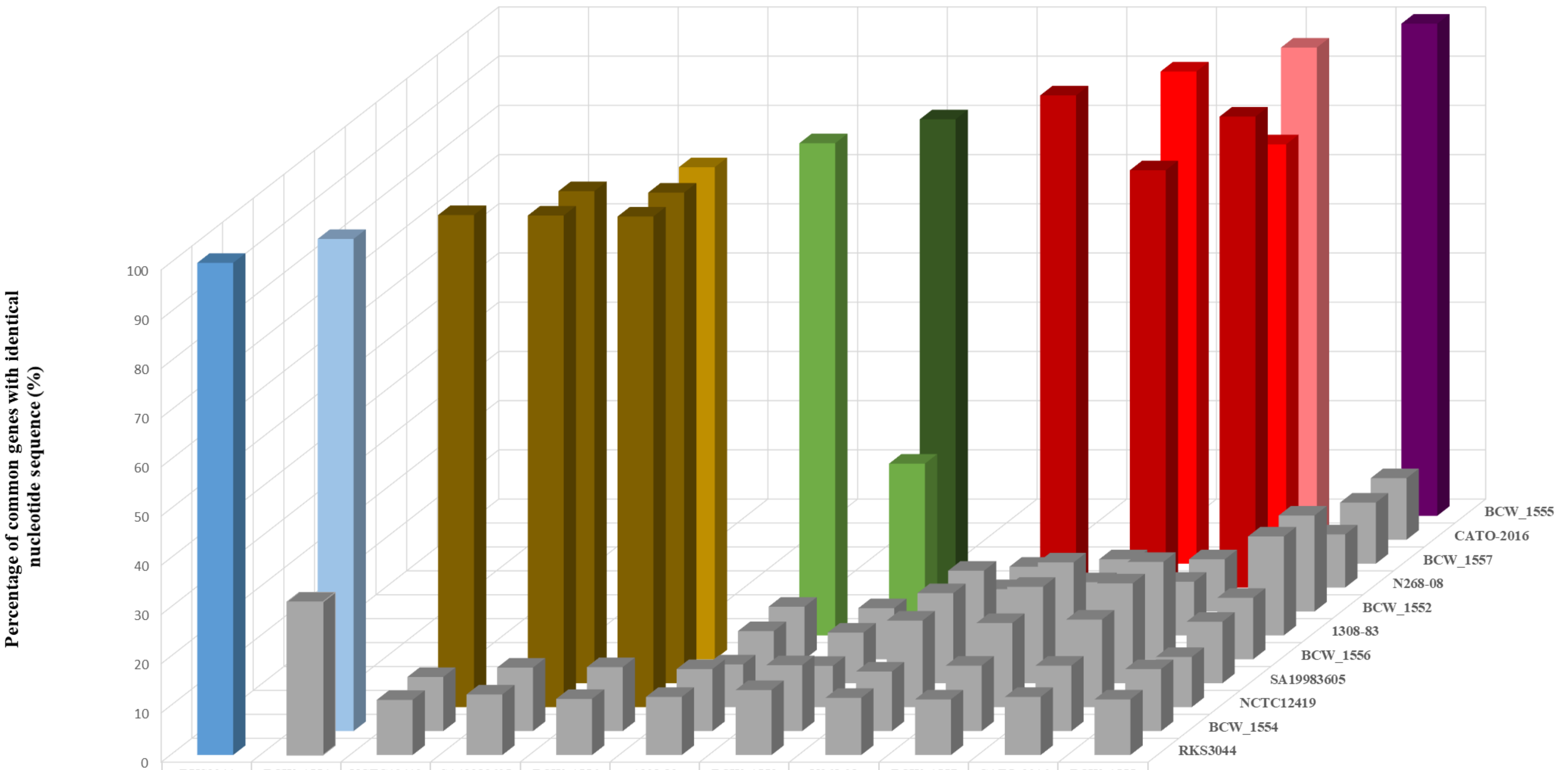

	RKS3044	BCW 1554	NCTC12419	SA19983605	BCW 1556	1308-83	BCW 1552	N268-08	BCW 1557	CATO-2016	BCW 1555
RKS3044	100	31.1	11.2	12.3	11.4	11.8	13.2	11.6	11.3	11.8	11.3
BCW_1554		100	11.0	12.9	13.0	12.6	13.4	12.1	13.3	13.3	12.6
NCTC12419			100	99.9	99.7	8.7	8.4	17.6	17.1	17.8	10.2
SA19983605				100	99.7	10.6	10.3	18.3	19.6	20.3	12.5
BCW_1556					100	10.7	10.4	18.0	19.7	19.8	12.5
1308-83						100	34.9	9.4	10.8	10.9	20.1
BCW_1552							100	9.1	10.6	10.6	19.5
N268-08								100	84.8	95.7	10.8
BCW_1557									100	85.2	12.4
CATO-2016										100	12.5
BCW_1555											100
